# Editorial: Novel biomaterials to improve the biological effects of electromagnetic and particle radiation

**DOI:** 10.3389/fbioe.2023.1357668

**Published:** 2024-01-05

**Authors:** Yun Sun, Xin Luan, Rui Liu, Arif Gulzar, Jiajun Fan

**Affiliations:** ^1^ Department of Research and Development, Shanghai Proton and Heavy Ion Center, Fudan University Cancer Hospital, Shanghai, China; ^2^ Shanghai Frontiers Science Center of TCM Chemical Biology, Institute of Interdisciplinary Integrative Medicine Research, Shanghai University of Traditional Chinese Medicine, Shanghai, China; ^3^ School of Chemistry and Molecular Engineering, Nanjing Tech University, Nanjing, China; ^4^ Centre for Advanced Imaging, Australian Institute for Bioengineering and Nanotechnology, The University of Queensland, Brisbane St Lucia, QLD, Australia; ^5^ Department of Biological Medicines and Shanghai Engineering Research Center of Immunotherapeutics, Fudan University School of Pharmacy, Shanghai, China

**Keywords:** radiation, hypothermia, radiodynamic therapy, photodynamic therapy, medical imaging

This editorial summarizes the contributions to the Frontiers Research Topic “*Novel biomaterials to improve the biological effects of electromagnetic and particle radiation*”, established under the Biomaterials section and appearing under the Frontiers in Bioengineering and Biotechnology journals.

Radiation, encompassing photons, microwaves, magnetism, and ionizing radiation such as X-rays, α, and γ-rays, as well as particle radiation, has emerged as a promising approach for disease treatment, offering advantages over surgical interventions due to reduced invasiveness. Scientists have made significant progress in the field by developing various therapeutic modalities, including photothermal therapy, photodynamic therapy, sonodynamic therapy, radiodynamic therapy, and particle therapy, exploiting the diverse properties of these radiation types. Among these modalities, radiotherapy based on ionizing radiation has proven particularly successful, with over 60% of tumor patients benefitting from its application. Nonetheless, tumor recurrence remains a persistent challenge. The primary obstacle lies in the constrained tolerance of surrounding normal tissue, which hinders the delivery of an effective treatment dose to the lesion. Furthermore, determining the optimal region within the lesion for treatment presents an additional dilemma.

To address these challenges, the development of biomaterials capable of absorbing radiation energy and generating additional free radicals to intensify lesion damage has emerged as a promising resolution, commonly referred to as dynamic therapy. Photodynamic therapy (PDT) stands out as the most renowned dynamic therapy. PDT has been subject to extensive research for over four decades, with numerous studies demonstrating significant anti-tumor efficacy and limited toxicity concerns. Initially anticipated to become a prominent therapeutic option for localized cancer treatment, PDT has not yet gained widespread acceptance as a standard-of-care approach. One major impediment is the technical difficulty in treating deep-seated and large tumors due to the limited penetration and variability of activating light within tissue (Dinakaran and Wilson).

Recent advancements, however, offer a novel therapeutic strategy known as radioPDT, facilitated by innovative nanomaterials. This approach integrates ionizing radiation energy, including high-energy X-rays, as a substitute for external light sources, thereby addressing the limitations associated with light penetration. Previous attempts to overcome this limitation involved using near-infrared light (Zhang et al., 2021). By leveraging nanomaterials, radioPDT combines the precise targeting and treatment depth/volume capabilities of radiation therapy with the favorable therapeutic index and biological advantages of PDT, without increasing toxicities (Dinakaran and Wilson). These nanomaterials typically incorporate high-Z metals to efficiently absorb X-ray photons and subsequently emit photons to excite photosensitizers. Notably, recent advancements have introduced novel single-molecular sensitizers capable of directly absorbing X-ray energy and catalyzing the production of free radicals, thereby achieving higher efficiency (Wang et al., 2020).

Thermal effects induced by various forms of radiation, such as photons, present a promising treatment modality with significant potential. In interventional procedures, clinicians employ lasers to generate thermal effects for tumor ablation. However, this approach has limitations concerning treatment depth. To address this challenge, magnetic nanoparticles (NPs) have emerged as potential candidates for hyperthermia cancer treatment, offering an efficient technique with minimal adverse effects on patients. Dubey et al. conducted a study where they substituted Bi cations in BiFe_0.95_Mn_0.05_O_3_ nanoparticles (BFM) with rare-earth (RE) elements, namely Nd, Gd, and Dy. This substitution resulted in notable changes in the morphology of the BFM nanoparticles, with Nd, Gd, and Dy doping leading to the alteration of the elliptical shape to rectangular and irregular hexagonal shapes, respectively. The introduction of RE dopants also conferred ferroelectric properties to the RE-BFM nanoparticles, exhibiting a larger piezoresponse compared to pristine BFO nanoparticles. Particularly, the incorporation of Gd led to a significant increase in the maximum magnetization of BFM by up to 550% at 300 K. In hyperthermia tests, a dispersion of NPs at a concentration of 3 mg/mL in water and agar demonstrated the ability to raise the temperature of the dispersion to approximately 39°C when subjected to an applied AC magnetic field of 80 mT (Dubey et al.).

In a separate study conducted by Chen Wang et al., they developed a synergistic magnetic and photothermal therapeutic nanoplatform. The researchers constructed mSiO2-SmCox (Sm/Co = 1:2) nanoparticles, which exhibited a highly dispersed and uniform morphology with an average diameter of approximately 73 nm. The photothermal conversion efficiency of the mSiO2-SmCox (Sm/Co = 1:2) nanoparticles was determined to be nearly 41%. Subsequent *in vitro* and *in vivo* evaluations of the anti-tumor efficacy of these nanoparticles demonstrated promising potentials for hyperthermia-induced tumor therapy owing to the combined magnetic and photothermal effects (Liang et al.).

When it comes to radiation therapy, a crucial question arises regarding which part of the lesion should be targeted for irradiation. In this context, medical imaging plays a pivotal role in guiding radiation therapy. Various imaging modalities, such as positron emission tomography (PET), magnetic resonance imaging (MRI), and computed tomography (CT), are employed for this purpose. Of particular interest is FAPI-PET imaging, which specifically targets cancer-associated fibroblasts known to promote tumor progression and exhibit radioresistance. FAPI-PET has been investigated in clinical trials to guide carbon ion radiotherapy. Herein, Wenjie Zhang et al. have reported on the development and evaluation of an 18F-labeled quinoline analogue as a potential FAP-targeted PET tracer that shows promise in penetrating the blood-brain barrier, enabling imaging in the brain (Zhang et al.).

In specific conditions such as colorectal cancer, real-time imaging tools like photoacoustic and ultrasound tomography provide unique insights during radiation therapy. These imaging techniques allow for visualization of the tumor and surrounding tissues, enabling clinicians to monitor the treatment response and make necessary adjustments in real-time. Jiaying Xiao et al. conducted a study on the cross-scale endoscopic imaging of rectal tumors using a combined photoacoustic/ultrasound tomography system along with wide-field optical microscopy. This multimodal system integrates several advantageous features, including deep penetration at the centimeter scale, multi-spectral imaging, cross-scale imaging capability, low system cost, and a 360° view within a single modality. The findings suggest that this proposed system can reliably identify the location of cancer invasion depth spectroscopically using indocyanine green. Additionally, the wide-field optical imaging mode effectively identifies tumor angiogenesis (Jiang et al.).

In summary, radiation therapy has undergone significant advancements in recent years, with researchers exploring innovative approaches to enhance treatment efficacy and minimize side effects. The development of nanomaterials and their integration into dynamic therapies such as radioPDT, as well as the utilization of magnetic and photothermal effects for hyperthermia-based tumor therapy, hold promise for improving patient outcomes. Additionally, the incorporation of advanced medical imaging techniques, including FAPI-PET, photoacoustic tomography, and ultrasound tomography, contributes to more precise treatment planning and real-time monitoring of radiation therapy. As research in this field continues, it is expected that these advancements will further enhance the effectiveness of radiation therapy and improve patient care.
